# Protective Effects of LSGYGP from Fish Skin Gelatin Hydrolysates on UVB-Induced MEFs by Regulation of Oxidative Stress and Matrix Metalloproteinase Activity

**DOI:** 10.3390/nu10040420

**Published:** 2018-03-28

**Authors:** Qingyu Ma, Qiuming Liu, Ling Yuan, Yongliang Zhuang

**Affiliations:** Yunnan Institute of Food Safety, Kunming University of Science and Technology, No. 727 South Jingming Road, Kunming 650500, Yunnan, China; mqy0323@hotmail.com (Q.M.); kgqml2012@163.com (Q.L.); ly15145551312@163.com (L.Y.)

**Keywords:** LSGYGP, UVB radiation, oxidant stress, matrix metalloproteinases, MAPK pathway

## Abstract

A previous study has shown that tilapia fish skin gelatin hydrolysates inhibited photoaging in vivo, and that, Leu-Ser-Gly-Tyr-Gly-Pro (LSGYGP) identified in the hydrolysate had a high hydroxyl radical scavenging activity. In this study, activities of LSGYGP were further evaluated using ultraviolet B (UVB)-induced mouse embryonic fibroblasts (MEFs). UVB irradiation significantly increased the intercellular reactive oxygen species (ROS) production and matrix metalloproteinases (MMPs) activities and decreased the content of collagen in MEFs. LSGYGP reduced the intercellular ROS generation in UVB-induced MEFs. Meanwhile, the decrease of superoxide dismutase (SOD) activity and the increase of malondiaidehyde (MDA) content were inhibited by LSGYGP. LSGYGP reduced MMP-1 and MMP-9 activities in a dose-dependent manner. Molecular docking simulation indicated that LSGYGP inhibited MMPs activities by docking the active sites of MMP-1 and MMP-9. Furthermore, LSGYGP also affected the intercellular phosphorylation of UVB-induced the mitogen-activated protein kinase pathway. LSGYGP could protect collagen synthesis in MEFs under UVB irradiation by inhibiting oxidative stress and regulating MMPs activities.

## 1. Introduction

Ultraviolet (UV) irradiation, including ultraviolet A (UVA) (315–400 nm) and ultraviolet B (UVB) (290–320 nm), can generate reactive oxygen species (ROS)/nitrogen species. Excessive oxidative stress can cause photoaging and even apoptotic or necrotic skin cell death [1]. Compared with UVA, UVB irradiation produces greater biological effects. UVB may pass through the epidermis, penetrate the upper part of the dermis, and induce the generation of excessive ROS [2]. The matrix metalloproteinases (MMPs) are a family of zinc-dependent endopeptidases and can degrade all components of extracellular matrix protein and connective tissues [3]. UV irradiation increases MMPs expression and protein kinases phosphorylation by mitogen-activated protein kinases (MAPK) pathway, which causes intercellular collagen destruction. Three distinct MAPK signal transduction pathways, including extracellular signal-regulated kinases (ERKs), c-Jun N-terminal kinases (JNKs), and p38, have important effects on signaling pathways that regulate MMPs expression [4].

ROS scavengers inhibit the intercellular UVB-induced damage by attenuating MMPs activity. The decline of ROS plays a role in photoaging activities and is possibly via the suppression of MMPs production [5,6]. In recent years, most studies focus on peptides derived from marine animal sources, including jumbo squid skin [7], pacific hake [8], spiny head croaker [9], sea cucumber [10], which possess good radical scavenging activities. Furthermore, previous studies showed that some peptides have good inhibitory photoaging activities. Zhuang et al. [11] showed that jellyfish collagen peptides could protect collagen fibers of photoaged mice skin. Chen et al. [12] indicated that hydrolysates from Pacific cod skin could block the up-regulation of MMPs expression in photoaging mice. Some studies showed that peptides from different sources have inhibitory MMPs activities in cells [13,14,15,16]. Chen et al. [13] reported that Chlorella-derived peptide had MMP-1 inhibitory activity in human skin fibroblast irradiated with UVB. Nguyen et al. [14] found that AELPSLPG had the MMPs inhibitory effects on the human fibrosarcoma. Ryu et al. [15] discovered that the peptide LEDPFDKDDWDNWK from sea horse inhibited the MMP expression in osteoblastic MG-63 cell. Lu et al. [16] reported that the hydrolysates from cod skin gelatin inhibited MMP-1 activity in UVB-induced mice fibroblasts, and two peptides (GEIGPSGGRGKPGKDGDAGPK and GFSGLDGAKGD) were identified.

A number of compounds can decrease MMPs activities by docking the active sites of MMPs [17]. A structure-activity relationship study is required to determine the interaction between peptides and MMP catalytic sites. It could be a powerful technology to use the structure-activity relationship for development of MMPs inhibitor using molecular docking simulation.

In our previous study, the effect of tilapia skin gelatin hydrolysates (TGHs) on UV-induced mice skin damages was determined. The results showed that TGHs regulated the UV-induced abnormal changes of antioxidant indicators in photoaging mice. TGHs could protect mice skin collagen fibers from UV irradiation damages. The action mechanisms of TGHs mainly involved antioxidant abilities and repaired to endogenous collagen synthesis. Fractionation of TGH led to the identification of peptide LSGYGP which displayed a high hydroxyl radical scavenging activity [18]. In this study, we aim to study the protective effects of LSGYGP on intercellular collagen formation, ROS generation and MMP levels in UVB-induced mouse embryonic fibroblasts (MEFs). The interaction between MMP active sites and LSGYGP was analyzed by molecular docking simulation. Furthermore, the regulation of LSGYGP on the intercellular phosphorylation of the MAPK signal pathway was studied.

## 2. Materials and Methods

### 2.1. Materials and Regents

LSGYGP was provided by Shanghai Synpeptide Co., Ltd. (Shanghai, China). Fetal bovine serum (FBS), dulbecco’s modified Eagle’s medium (DMEM), phosphate buffered saline (PBS), and trypsin (0.25%) were purchased from Gibco (New York, NY, USA). 3-(4,5 dimethylthiazol-2-yl)-2,5-diphenyltetrazolium bromide (MTT) was provided from Sigma Chemical Co. (St. Louis, MO, USA). 2′,7′-dichlorodihydrofluorescein diacetate ROS assay kit was purchased from Beyotime Biotechnology Co., Ltd. (Shanghai, China). Superoxide dismutase (SOD) and malondialdehyde (MDA) commercial kits were provided from Nanjing Jiancheng Institute of Biotechnology (Nanjing, China). The ELISA kits of Collagen I, MMP-1, MMP-9, p-JNK, p-ERK, and p-p38 were purchased from R&D (Systems Inc., Minneapolis, MN, USA).

### 2.2. Cell Culture

MEFs were isolated from the dermis of ICR fetal mice according to the previous method [16,19] and cultured in 5% CO_2_ at 37 °C in DMEM with FBS (10%, *v*/*v*), penicillin (100 U/mL), and streptomycin (100 mg/L). The generations 5–7 of MEFs were selected for further assays.

### 2.3. UVB Irradiation and Cell Viability Assay

MEFs (2 × 10^4^ cells/well) were plated in 96-well plates containing DMEM with FBS (10%, *v*/*v*) and incubated in 5% CO_2_ at 37 °C for 24 h. MEFs were further incubated in culture medium with LSGYGP (20, 40 and 80 μM) for 24 h, and then the cells were placed in a thin layer of PBS. MEFs-treated were exposed to UVB using two lamps (Beijing Zhongyiboteng-tech Co., Ltd., Beijing, China) that emitted UVB peaking at 313 nm, which delivered uniform irradiation at a distance of 30 cm. MEFs were irradiated by UVB with a dose of 30 mJ/cm^2^, which was determined with a UV-313 radiometer (Photoelectric Instrument Factory of Beijing Normal University, China). The PBS was replaced by the culture medium with LSGYGP and incubated at 37 °C for an additional 12 h. Finally, MEFs and culture supernatants were obtained for further study. The normal control (NC) group was cultured at the same condition without UVB irradiation and LSGYGP, and the model control (MC) group was exposed to UVB irradiation without LSGYGP.

Cell viability was evaluated with MTT assay [20]. The collected cells were treated with 150 μL MTT reagents (0.5 mg/mL) for 4 h at 37 °C. Subsequently, the MTT reagent was removed, and the amount of MTT formazan was solubilised with 150 μL of dimethyl sulfoxide (DMSO). The absorbance of each well was measured using ELISA (Spectra Max M5; Molecular Devices, Winooski, VT, USA) at 570 nm.

### 2.4. Determination of Generated Intracellular ROS

The levels of intracellular ROS were determined with fluorescence assay using a DCFH-DA ROS assay kit [21]. MEFs (2 × 10^4^ cells/well) were plated in 12-well plates, and UVB irradiation was the same with that in Section 2.3. The collected cells were resuspended in freshly prepared serum-free medium that contained 10 μM DCFH-DA at 37 °C for 20 min in the dark. MEFs were then harvested and washed with PBS. The oxidative formation of DCFH-DA via intracellular ROS was immediately examined at λex of 485 nm and λem of 535 nm using a flow cytometer (Guava easyCyte 6-2L; EMD Millipore, Hayward, CA, USA). The result was expressed by relative content compared with that of the NC group.

### 2.5. Analysis of Intracellular SOD and MDA

MEFs (2 × 10^4^ cells/well) were plated in 12-well plates, and the UVB irradiation was the same with that in Section 2.3. The collected cells resuspended in PBS and lysates were prepared under ultrasonic and centrifuged. The supernatants were quantified using BCA protein assay. Intracellular SOD activity and MDA content was measured by the respective kits, and they were expressed by U/mg protein (prot) and nM/mg prot.

### 2.6. ELISA Assays of Collagen I, MMP-1, and MMP-9

MEFs (2 × 10^4^ cells/well) were plated in 6-well plates, and the UVB irradiation was same with that in Section 2.3. The culture supernatants were collected and the collagen I content and the MMP-1 and MMP-9 activities were determined by ELISA, which were performed according to the manufacturer’s protocol of the respective kits.

### 2.7. Molecular Docking Analysis

The three-dimensional structures of MMP-1 (966c.pdb) and MMP-9 (1gkc.pdb) were obtained from the Protein Data Bank. Water molecules were removed, and the cofactors zinc and chloride atoms were retained in the MMPs model [22]. The structure of LSGYGP was built by using SYBYL2.1.1 software (Tripos Associates, St. Louis, MO, USA). Hydrogen atoms were added to crystal structures 966c and 1gkc in SYBYL. The docking of LSGYGP onto the active sites of MMP-1 and MMP-9 was performed by molecular visualization. T-score, C-score, hydrogen bond, and distance were calculated by SYBYL [22].

### 2.8. ELISA Analysis of JNK, ERK, and p38

MEFs (2 × 10^4^ cells/well) were plated in 6-well plates, and the UVB irradiation was the same with that in Section 2.3. The collected cells were lysed by rapid freezing and thawing, and the contents of p-ERK, p-JNK, and p-p38 were tested by respective kits, which were operated according to the manufacturer’s protocol.

### 2.9. Statistical Analysis

The outcomes for cell viability, collagen production, intercellualar SOD activity and MDA content, ROS production, MMPs activity and phosphorylation of MAPK were presented as the mean value with standard deviation. The significant differences between different groups were analyzed with multiple comparison test using SPSS (version 17.0, IBM Inc., Chicago, IL, USA). The statistical differences were considered significant at *p* < 0.05 and the significant differences were expressed using different lowercase letters. The molecular docking visualization, T-score, C-score, hydrogen bond and distance were evaluated by SYBYL 2.1.1 software (Tripos, Associaties, Inc., St. Louis, MO, USA).

## 3. Results

### 3.1. Cell Viability

**Figure 1:**
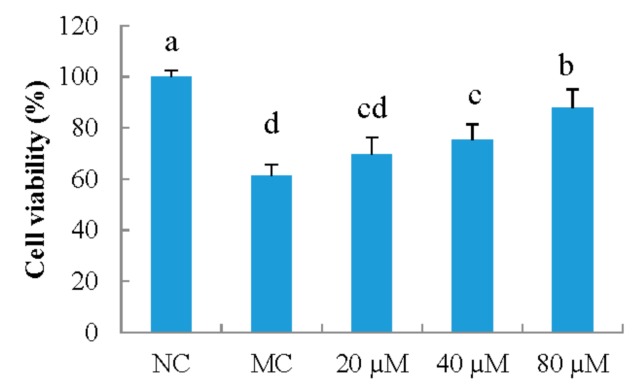
The effect of LSGYGP with different concentrations (20, 40, 80 μM) on cell viability in mouse embryonic fibrolasts (MEFs) ultraviolet B (UVB)-induced. Bar values with different letters were significant difference (*p* < 0.05). NC: nomal control; MC: model control.

As shown in Figure 1, the effect of LSGYGP on the viability of MEFs was determined. UVB irradiation significantly decreased the viability of MEFs compared with that of the NC group (*p* < 0.05). LSGYGP had high protective effect on cell viability against UV irradiation, and the effect of LSGYGP was in a dose-dependent manner. LSGYGP with a dose of 40 μM had significantly different with MC group (*p* < 0.05).

### 3.2. Collagen I

**Figure 2:**
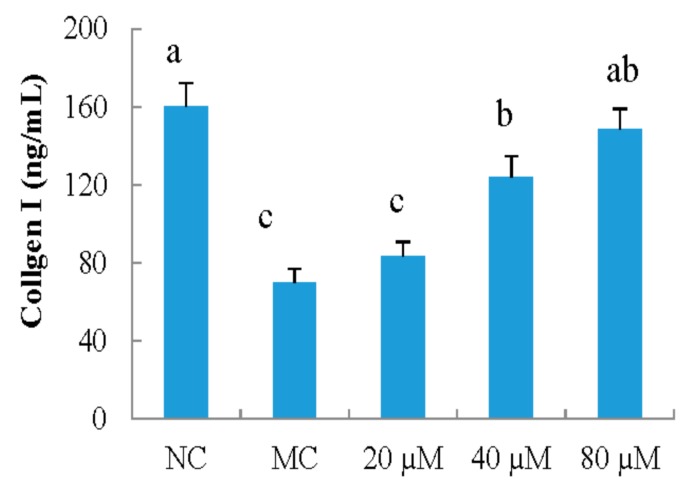
The effect of LSGYGP on collagen production in mouse embryonic fibrolasts (MEFs) ultraviolet B (UVB)-induced. Bar values with different letters were significant difference (*p* < 0.05).

Figure 2 shows different concentrations of LSGYGP inhibiting decrease in collagen content in MEFs UVB-induced. Compared with that of NC group, the collagen content of MC group significantly decreased by 56.2% (*p* < 0.05). LSGYGP potently increased the collagen production in MEFs, and the effect was in a dose-dependent manner. Intracellular collagen contents were significantly increased more than twice at LSGYGP with the concentration of 80 μM compared with that of MC group.

### 3.3. Antioxidant Indicators

**Figure 3:**
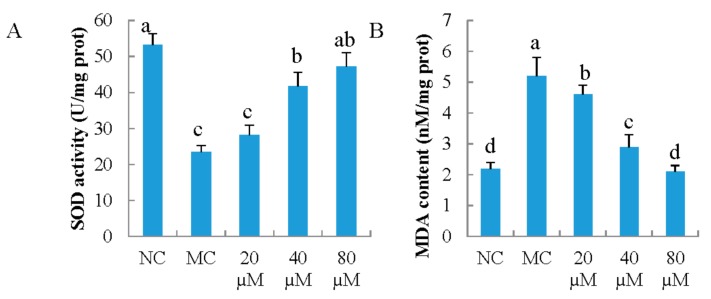
The effect of LSGYGP on intercellular superoxide dismutase (SOD) activity (**A**) and malondiaidehyde (MDA) content (**B**) in mouse embryonic fibrolasts (MEFs) ultraviolet B (UVB)-induced. Bar values with different letters were significant difference (*p* < 0.05).

As shown in Figure 3A, the activities of SOD enzymes were decreased by UVB irradiation, and the activities of MC group decreased by 56.0% compared with that of NC group. LSGYGP protected intercellular SOD activities against the UVB damages in a dose-dependent manner. LSGYGP with a dose of 40 μM was significantly higher than that in MC group (*p* < 0.05).

The results (Figure 3B) showed that UVB irradiation caused the MDA content in MC group to increase by 136.4%, compared with that of NC group (*p* < 0.05). MDA increase was significantly inhibited by LSGYGP (*p* < 0.05), and the inhibitory ability of LSGYGP was in a dose-dependent manner. Compared with NC group, it had no significant difference with LSGYGP at a dose of 80 μM (*p* > 0.05).

### 3.4. ROS Generation

**Figure 4:**
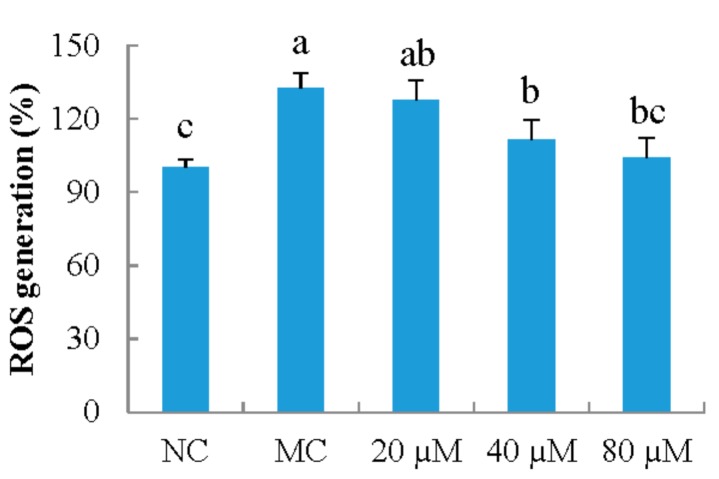
The effect of LSGYGP on intercellular reactive oxygen species (ROS) production in mouse embryonic fibrolasts (MEFs) ultraviolet B (UVB)-induced. Bar values with different letters were significant difference (*p* < 0.05).

A previous study showed excessive ROS generation from UVB irradiation can induce photoaging [5]. In this study, the effect of LSGYGP on UVB-induced ROS production in MEFs was evaluated. The results showed that UVB irradiation markedly increased ROS production by 32.4%, compared with that in NC group (Figure 4). LSGYGP regulated the intercellular ROS generation under UVB irradiation, and this inhibitory activity was in a dose-dependent manner.

### 3.5. Intercellular MMP-1 and MMP-9 Activity

**Figure 5:**
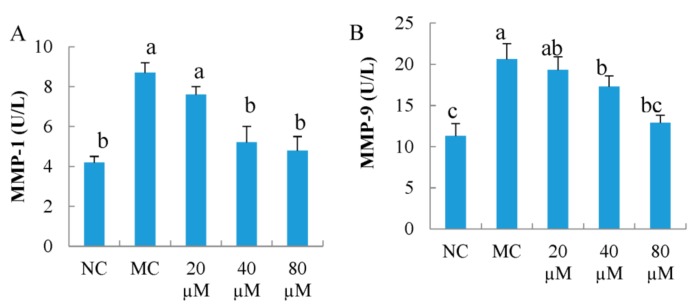
The effect of LSGYGP on intercellular matrix metalloprpteinases (MMP)-1 (**A**) and MMP-9 (**B**) activity in mouse embryonic fibrolasts (MEFs) ultraviolet B (UVB)-induced. Bar values with different letters were significant difference (*p* < 0.05).

As shown in Figure 5, UVB irradiation dramatically increased the activities of MMP-1 and MMP-9 in MEFs (*p* < 0.05). LSGYGP significantly decreased the up-regulated activities of MMP-1 and MMP-9 (*p* < 0.05) in a dose-dependent manner. Meanwhile, the LSGYGP with 40 μM group significantly inhibited of MMP-1 and MMP-9 activities (*p* < 0.05), compared with that of the MC group.

### 3.6. Molecular Docking

**Figure 6:**
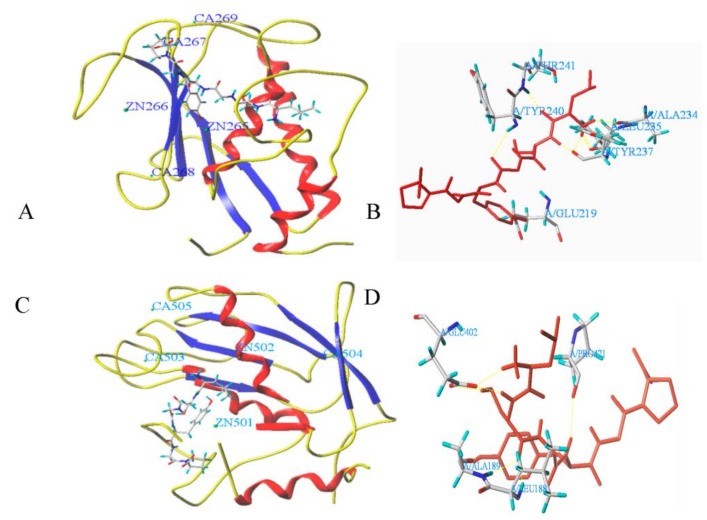
The molecular docking between LSGYGP and matrix metalloprpteinases (MMP). (**A**) and (**B**): docking of LSGYGP and MMP-1; (**C**) and (**D**): docking of LSGYGP and MMP-9.

Figure 6 shows the binding of LSGYGP at the active sites of MMP-1 and MMP-9. LSGYGP entered the narrow S_1_′-subpocket and created strong interactions with the enzymes. The docking simulation of LSGYGP at the MMP-1 and MMP-9 active sites in the presence of Zn(II) showed the best docking pose, and the total scores were 7.30 and 8.53 (Table 1), respectively. Table 1 indicated the all possible direct hydrogen bond interactions. After docking, the peptide LSGYGP had eight hydrogen bonds with MMP-1 residues, including Glu219, Tyr240, Thr241, Tyr237, and Leu235. Seven hydrogen bonds of MMP-9 and LSGYGP were formed with Pro421, Leu188, Ala189, and Glu402, and four hydrogen bonds were formed between LSGYGP and Glu402 of MMP-9. The short 1.98 Å and 2.02 Å hydrogen bonds were found between LSGYGP and Zn(II) of MMP-1 and MMP-9, respectively.

### 3.7. The Phosphorylation of ERK, JNK, and p38 in MEFs

**Figure 7:**
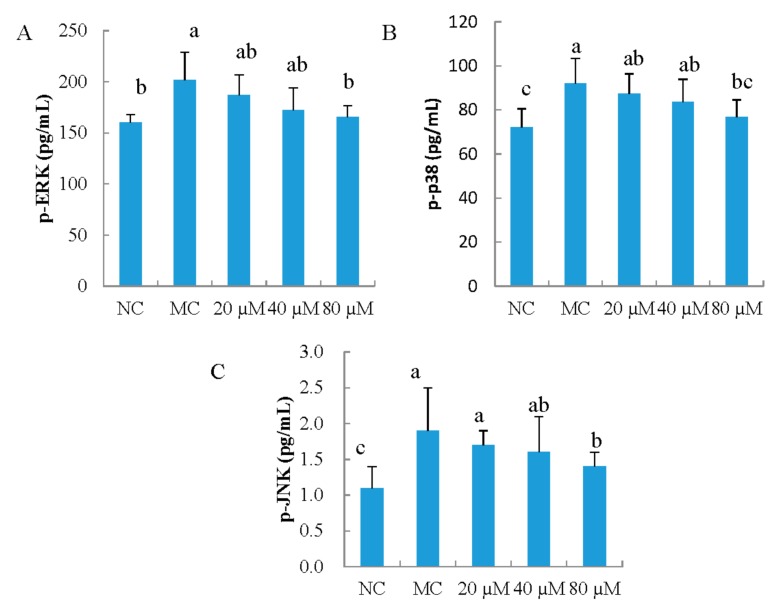
The effect of LSGYGP on the phosphorylation of extracellular signal-regulated kinases (ERK) (**A**), p38 (**B**) and jun N-terminal kinases (JNK) (**C**) of mitogen-activated protein kinases (MAPK) signaling pathway in mouse embryonic fibrolasts (MEFs) ultraviolet B (UVB)-induced. Bar values with different letters were significant difference (*p* < 0.05).

In this study, the effect of LSGYGP via the MAPK pathway on the up-regulated expressions of MMPs was investigated. As shown in Figure 7, UVB irradiation caused a significant increase in p-ERK, p-JNK, and p-p38 levels in MEFs (*p* < 0.05). LSGYGP regulated the phosphorylation of ERK, JNK, and p38 in a dose-dependent manner. A significant difference occurred in the phosphorylation of ERK, JNK, and p38 levels between LSGYGP with 80 μM group and MC group (*p* < 0.05). Our results indicated that LSGYGP could inhibit the phosphorylation of ERK, JNK, and p38 in the MAPK signaling pathway.

## 4. Discussion

In photoaging damage, the decrease of collagen formation is a main characteristic feature. UVB-irradiation increases the production of ROS within the cells. ROS plays a significant role in the declension of collagen synthesis [6]. In our previous study, TGHs was shown to inhibit photoaging damage changes in mice UV-induced model, and successive separation of TGHs led to the identification of LSGYGP that displayed a strong hydroxyl radical scavenging activity in vitro [18]. In this study, collagen in MEFs was severely destroyed by UVB irradiation, and the collagen content decreased by 47.9% after 30 mJ/cm^2^ of UVB radiation. LSGYGP could significantly inhibit UVB-induced collagen decrease. The previous studies indicated UVB irradiation affected many biological functions involving the synthesis of MMPs [2]. MMPs can destroy collagen synthesis in the skin dermis. Concerning the action mechanisms of decreasing MMPs levels, the blocking of the MMP secretion and bonding of enzyme activity sites may be mainly involved in inhibiting the catalytic reaction of MMPs. Several studies reported peptides with Leu and Pro had high inhibitory MMPs activities [14,23], and LSGYGP was in accordance to this characteristic. To understand the potential mechanisms of the LSGYGP effects on photoaging, we study whether LSGYGP regulates oxidant stress and inhibits MMPs using MEFs UVB-induced model. Furthermore, the effect of LSGYGP of direct docking active sites of MMPs was evaluated.

Intracellular ROS content directly reflects reducing power in cells [6]. Compared with normal MEFs, UVB irradiation significantly increased intracellular ROS content in this study. LSGYGP can inhibit the increase of UVB-induced ROS significantly. In normal physiological conditions, the formation of oxidants is balanced by efficient removal of antioxidative enzymes, including SOD, CAT, and GSH-Px. SOD is the initial enzyme in the enzymatic defense system in vivo and converts superoxide radicals into hydrogen peroxide [24]. The activities of intracellular SOD were decreased by UVB irradiation-induced ROS. LSGYGP can inhibit the decrease of SOD activities. Meanwhile, intercellular ROS UVB-induced enhances the oxidative stress of skin cell membrane lipids, which causes a large amount of lipid peroxidation product MDA. This process consequently induces skin photoaging [25]. In this study, the MDA UVB-induced was significantly decreased by LSGYGP. Therefore, LSGYGP could decrease intercellular lipid peroxidation damages UVB-induced and alleviate photo-oxidative stress of collagen production through regulating the activities of antioxidant enzymes. LSGYGP could affect the collagen metabolism of photoaging cells by the alleviation of intercellular oxidative stress.

The MMP family can cause the degradation of skin collagen and lead to photoaging. Currently, over 20 types of MMPs were divided into collagenases, matrilysins, gelatinases, stromelysins, and membrane-type MMPs, according to their function [26]. MMP-1 belongs to the collagenase and degrades collagen I, which is a major composition in the skin dermis. The increase of MMP-1 expression can cause skin collagen damage. Collagen is the richest protein in the dermal connective tissue and collagen is broken in pieces by MMP-1 [27]. Collagen fragments are further hydrolyzed via other MMPs, such as MMP-2 (gelatinase A) and MMP-9 (gelatinase B). A variety of naturally occurring agents, which have inhibitory MMPs ability, have been widely used to prevent photoaging [26]. Thus, finding of MMP-1 and MMP-9 inhibitors is considered as a promising strategy to increase the type I procollagen production for skin photoaging therapies. In this study, the cell viability and the activities of MMP-1 and MMP-9 of UVB-induced MEFs were examined. UVB irradiation could significantly attenuate MEFs death and increase the activities of intercellular MMP-1 and MMP-9 of MEFs by UV radiation. LSGYGP could regulate the abnormal changes during UVB irradiation. 

Previous studies showed that the production of excessive ROS could affect the levels of MMPs [6]. Thus, the effect of LSGYGP on MMPs was due to its scavenging ROS ability. We are interested in finding out whether the LSGYGP has any direct interaction in the active sites of MMP-1 and MMP-9 proteins, which can suppress MMP-1 and MMP-9 activities. The structures of MMPs are similar, and the main differences occur in the S_1_′ pocket. The S_1_′ pocket of MMP-1 is short and narrow, and the S_1_′ specificity site in MMP-9 is described as tunnel [28,29]. The molecular docking simulation could be used as a tool to evaluate the mechanisms of molecular interactions between MMPs and LSGYGP. Our results showed that LSGYGP could enter the S_1_′-subpocket of MMP-1 and MMP-9 and give strong interactions with the enzymes. The docking simulation of LSGYGP at the MMP-1 and MMP-9 active sites with Zn(II) displayed the best docking pose (Figure 6) with high T-score and right C-score. Hydrogen bonds interaction force is important in stabilizing the docking complex and catalytic reactions [28]. After docking, the peptide LSGYGP formed eight and seven hydrogen bonds with MMP-1 and MMP-9 residues, respectively. Previous studies showed that Tyr 240 and Thr 241 were key hydrophobic and polar amino acids of the S_1_ pocket of MMP-1 [22], and the essential glutamic acid residue (402) was key to the catalytic center of MMP-9 [22,29]. Our results showed that LSGYGP could form hydrogen bonds with the Tyr 240 and Thr 241 of MMP-1 and the Glu 402 of MMP-9, respectively. The results suggested that LSGYGP effectively interacted with the active sites of MMPs, which enhanced its inhibitory MMPs activities. Zn(II) at the MMPs active sites often plays an important part for MMPs activity [22,28]. The distances of the oxygen atoms of LSGYGP and Zn(II) were determined in this study. The short 1.98 Å and 2.02 Å were found in MMP-1 and MMP-9, suggesting that LSGYGP could effectively interact with the active sites of MMPs and inhibit the enzymatic activity. Furthermore, LSGYGP had a hydrophobic amino acid (Pro) at the carboxy terminal and the aromatic group in Tyr, and the side chains of Pro and the aromatic group of Tyr may accommodate with the S_1_′ pocket of MMP-1 and MMP-9 and increase inhibitory MMPs potential.

Exposure to UVB irradiation has been shown to stimulate the phosphorylation of protein kinases by the MAPK pathway. The phosphorylation of MAPK pathway directly activates the transcription factor AP-1, which can increase the expression of MMPs. Thus, MAPK pathways play an important role in regulating MMP expression [30]. MAPKs contain three general classes, ERK, JNK, and p38 kinases. The phosphorylation of JNK can activate phospho-c-Jun. Phospho-c-Jun may form AP-1 and further enhance the expressions of MMPs. In addition, the phosphorylation of ERK activates the transcription of c-Fos and c-Jun, which are also known to form AP-1. In this study, we investigated whether the MMPs via the MAPK signaling pathway were influenced by LSGYGP. Our data suggested that LSGYGP regulated the phosphorylation of ERK, p38, and JNK in the MAPK pathway, thereby inhibiting the up-regulation of UVB-induced MMP expression. Therefore, the effect of LSGYGP on the MAPK signaling pathway may be one of the key mechanisms to suppress the up-regulated expression of MMPs caused by UVB irradiation.

## 5. Conclusions

LSGYGP showed high hydroxyl radical scavenging activities in our previous study. We further study the effects of LSGYGP on intercellular MMP activity and oxidative stress in UVB-induced MEFs. Our results indicated, for the first time, that the photoaging signs inhibitory activities of LSGYGP resulted from suppressing intercellular ROS generation, regulating intercellular reducing power, and decreasing the UVB-mediated expression of MMPs, thereby blocking the MAPK pathways. Furthermore, LSGYGP could bind to the active sites of MMP-1 and MMP-9, decrease their activities, and inhibit collagen degradation. Thus, LSGYGP may be a potential agent in tackling the signs of photoaging.

## Figures and Tables

**Table 1:** The data of molecular docking between LSGYGP and matrix metalloprpteinases (MMP).

LSGYGP	T-Score	C-Score	Hydrogen Bond Number	Distance (Å)
MMP-1	7.30	5	8	Glu219: 2.06; Tyr240: 2.62; Thr241: 2.02; Tyr237: 2.10/2.05; Leu235: 1.90/1.81/2.58 Zn265: 1.98
MMP-9	8.53	4	7	Pro421: 2.33; Leu188: 1.65; Ala189: 2.27; GLU402: 2.44/2.27/1.93/2.31; Zn1450: 2.02

## References

[1] Fan J., Zhuang Y., Li B. (2013). Effects of collagen and collagen hydrolysate from jellyfish umbrella on histological and immunity changes of mice photoaging. Nutrients.

[2] Leirós G.J., Kusinsky A.G., Balañá M.E., Hagelin K. (2017). Triolein reduces MMP-1 upregulation in dermal fibroblasts generated by ROS production in UVB-irradiated keratinocytes. J. Dermatol. Sci..

[3] Mohamed M.A., Jung M., Lee S.M., Lee T.H., Kim J. (2014). Protective effect of Disporum sessile D. Don extract against UVB-induced photoaging via suppressing MMP-1 expression and collagen degradation in human skin cells. J. Photochem. Photobiol. B.

[4] Chiang H.M., Lin T.J., Chiu C.Y., Chang C.W., Hsu K.C., Fan P.C., Wena K.C. (2011). *Coffeaarabica* extract and its constituents prevent photoaging by suppressing MMPs expression and MAP kinase pathway. Food Chem. Toxicol..

[5] Hong Y.F., Lee H., Jung B.J., Jang S., Chung D.K., Kim H. (2015). Lipoteichoic acid isolated from *Lactobacillus plantarum* down-regulates UV-induced MMP-1 expression and up-regulates type I procollagen through the inhibition of reactive oxygen species generation. Mol. Immunol..

[6] Muller F.L., Lustgarten M.S., Jang Y., Richardson A., Van Remmen H. (2007). Trends in oxidative aging theories. Free Radic. Biol. Med..

[7] Mendis E., Rajapakse N., Byun H.G., Kim S.K. (2005). Investigation of jumbo squid (*Dosidicus gigas*) skin gelatin peptides for their in vitro antioxidant effects. Life Sci..

[8] Cheung I.W.Y., Cheung L.K.Y., Tan N.Y., Li-Chan E.C.Y. (2012). The role of molecular size in antioxidant activity of peptide fractions from Pacific hake (*Merlucciu sproductus*) hydrolysates. Food Chem..

[9] Song R., Shi Q., Gninguue A., Wei R., Luo H. (2017). Purification and identification of a novel peptide derived from by-products fermentation of spiny head croaker (*Collichthys lucidus*) with antifungal effects on phytopathogens. Process Biochem..

[10] Zhou X., Wang C., Jiang A. (2012). Antioxidant peptides isolated from sea cucumber *Stichopus Japonicus*. Eur. Food Res. Technol..

[11] Zhuang Y., Hou H., Zhao X., Zhang Z., Li B. (2009). Effects of collagen and collagen hydrolysate from jellyfish (*Rhopilema esculentum*) on mice skin photoaging induced by UV irradiation. J. Food Sci..

[12] Chen T., Hou H., Fan Y., Wang S., Chen Q., Si L., Li B. (2016). Protective effect of gelatin peptides from pacific cod skin against photoaging by inhibiting the expression of MMPs via MAPK signaling pathway. J. Photochem. Photobiol. B.

[13] Chen C.L., Liou S.F., Chen S.J., Shih M.F. (2011). Protective effects of Chlorella-derived peptide on UVB-induced production of MMP-1 and degradation of procollagen genes in human skin fibroblasts. Regul. Toxicol. Pharm..

[14] Nguyen V.T., Qian Z.J., Ryu B.M., Kim K.N., Kim D., Kim Y.M., Jeon Y.J., Park W.S., Choi I.W., Kim G.H. (2013). Matrix metalloproteinases (MMPs) inhibitory effects of an octameric oligopeptide isolated from abalone Haliotis discus hannai. Food Chem..

[15] Ryu B.M., Qian Z.J., Kim S.K. (2010). Purification of a peptide from seahorse, that inhibits TPA-induced MMP, iNOS and COX-2 expression through MAPK and NF-kappaB activation, and induces human osteoblastic and chondrocytic differentiation. Chem. Biol. Int..

[16] Lu J., Hou H., Fan Y., Yang T., Li B. (2017). Identification of MMP-1 inhibitory peptides from cod skin gelatin hydrolysates and the inhibition mechanism by MAPK signaling pathway. J. Func. Foods.

[17] Parthasarathy A., Gopi V., Devi K.M.S., Balaji N., Vellaichamy E. (2014). Aminoguanidine inhibits ventricular fibrosis and remodeling process in isoproterenol-induced hypertrophied rat hearts by suppressing ROS and MMPs. Life Sci..

[18] Sun L., Zhang Y., Zhuang Y. (2013). Antiphotoaging effect and purification of an antioxidant peptide from tilapia (*Oreochromis niloticus*) gelatin peptides. J. Func. Foods.

[19] Ren S.W., Li J., Wang W., Guan H.S. (2010). Protective effects of j-ca3000 + CP against ultraviolet-induced damage in HaCaT and MEF cells. J. Photochem. Photobiol. B..

[20] Huang S., Ma Y., Sun D., Fan J., Cai S. (2017). In vitro DNA damage protection and anti-inflammatory effects of Tartary buckwheats (*FagopyrumtataricumL. Gaertn*) fermented by filamentous fungi. Int. J. Food Sci. Technol..

[21] Zhuang Y., Ma Q., Guo Y., Sun L. (2017). Protective effects of rambutan (*Nephelium lappaceum*) peel phenolics on H_2_O_2_-induced oxidative damages in HepG2 cells and d-galactose-induced aging mice. Food Chem. Toxicol..

[22] Sun L., Liu Q., Fan J., Li X., Zhuang Y. (2018). Purification and Characterization of Peptides Inhibiting MMP-1 Activity with C Terminate of Gly-Leu from Simulated Gastrointestinal Digestion Hydrolysates of Tilapia (*Oreochromis niloticus*) Skin Gelatin. J. Agric. Food Chem..

[23] Roomi M.W., Ivanov V., Niedzwiecki A., Rath M. (2004). Synergistic antitumor effect of ascorbic acid, lysine, proline, and epigallocatechin gallate on human fibrosarcoma cells HT1080. Ann. Cancer Res. Ther..

[24] Hou H., Li B., Zhao X., Zhuang Y., Ren G., Yan M., Zhang X., Chen L., Fan Y. (2009). The effect of pacific cod (*Gadusmacrocephalus*) skin gelatin polypeptides on UV radiation induced skin photoaging in ICR mice. Food Chem..

[25] Ma Q., Guo Y., Sun L., Zhuang Y. (2017). Anti-diabetic effects of phenolic extract from rambutan peels (*Nephelium lappaceum*) in high-fat diet and streptozotocin-induced diabetic mice. Nutrients.

[26] Fanjul-Fernández M., Folgueras A.R., Cabrera S., López-Otín C. (2010). Matrix metalloproteinases: Evolution, gene regulation and functional analysis in mouse models. Biochim. Biophys. Acta.

[27] You G.E., Jung B.J., Kim H.R., Kim H.G., Kim T.R., Chung D.K. (2013). Lactobacillus sakei lipoteichoic acid inhibits MMP-1 induced by UVA in normal dermal fibroblasts of human. J. Microbiol. Biotechnol..

[28] Yuan H., Lu W., Wang L., Shan L., Li H., Huang J., Sun Q., Zhang W. (2013). Synthesis of derivatives of methyl rosmarinate and their inhibitory activities against matrix metalloproteinase-1 (MMP-1). Eur. J. Med. Chem..

[29] Sarkar J., Nandy S.K., Chowdhury A., Chakraborti T., Chakraborti S. (2016). Inhibition of MMP-9 by green tea catechins and prediction of their interaction by molecular docking analysis. Biomed. Pharm..

[30] Chao W., Deng J., Huang S., Li P., Liang Y., Huang G. (2017). 3, 4-dihydroxybenzalacetone attenuates lipopolysaccharide-induced inflammation in acute lung injury via down-regulation of MMP-2 and MMP-9 activities through suppressing ROS-mediated MAPK and PI3K/AKT signaling pathways. Int. Immunopharm..

